# Light-Emitting-Diode-Based Multispectral Photoacoustic Computed Tomography System

**DOI:** 10.3390/s19224861

**Published:** 2019-11-08

**Authors:** Sumit Agrawal, Christopher Fadden, Ajay Dangi, Xinyi Yang, Hussain Albahrani, Neilesh Frings, Sara Heidari Zadi, Sri-Rajasekhar Kothapalli

**Affiliations:** 1 Department of Biomedical Engineering, Pennsylvania State University, University Park, State College, PA 16802, USA; sua347@psu.edu (S.A.); axd571@psu.edu (A.D.); xxy5131@psu.edu (X.Y.); hua17@psu.edu (H.A.); neilfrings@gmail.com (N.F.); sxh5604@psu.edu (S.H.Z.); 2 Department of Electrical Engineering, Pennsylvania State University, University Park, State College, PA 16802, USA; czf41@psu.edu; 3 Penn State Cancer Institute, Pennsylvania State University, Hershey, PA 17033, USA; 4 Graduate Program in Acoustics, The Pennsylvania State University, University Park, PA 16802, USA

**Keywords:** photoacoustic computed tomography, light-emitting diodes (LED), oxygen saturation, functional imaging, molecular imaging

## Abstract

Photoacoustic computed tomography (PACT) has been widely explored for non-ionizing functional and molecular imaging of humans and small animals. In order for light to penetrate deep inside tissue, a bulky and high-cost tunable laser is typically used. Light-emitting diodes (LEDs) have recently emerged as cost-effective and portable alternative illumination sources for photoacoustic imaging. In this study, we have developed a portable, low-cost, five-dimensional (*x, y, z, t,* λ) PACT system using multi-wavelength LED excitation to enable similar functional and molecular imaging capabilities as standard tunable lasers. Four LED arrays and a linear ultrasound transducer detector array are housed in a hollow cylindrical geometry that rotates 360 degrees to allow multiple projections through the subject of interest placed inside the cylinder. The structural, functional, and molecular imaging capabilities of the LED–PACT system are validated using various tissue-mimicking phantom studies. The axial, lateral, and elevational resolutions of the system at 2.3 cm depth are estimated as 0.12 mm, 0.3 mm, and 2.1 mm, respectively. Spectrally unmixed photoacoustic contrasts from tubes filled with oxy- and deoxy-hemoglobin, indocyanine green, methylene blue, and melanin molecules demonstrate the multispectral molecular imaging capabilities of the system. Human-finger-mimicking phantoms made of a bone and blood tubes show structural and functional oxygen saturation imaging capabilities. Together, these results demonstrate the potential of the proposed LED-based, low-cost, portable PACT system for pre-clinical and clinical applications.

## 1. Introduction

Photoacoustic computed tomography (PACT) is a nonionizing and noninvasive hybrid imaging modality that provides three-dimensional (3-D) optical-absorption-based functional and molecular contrast images of living subjects with high spatial resolution using ultrasound detection [1,2,3,4]. It relies on the photoacoustic effect, which is the generation of broadband ultrasound waves by the tissue chromophores that absorb short light pulses and undergo thermoelastic expansion. The generated ultrasound pressure waves propagate to ultrasound transducer detectors surrounding the living subject and are converted into volumetric photoacoustic images using image reconstruction methods. PACT has been demonstrated to map a wide range of intrinsic tissue chromophores, such as oxy- and deoxy forms of hemoglobin, melanin, and water, as well as extrinsic contrast agents, such as small molecules (e.g., methylene blue) and nanoparticles [5,6,7,8]. In particular, the capability to provide label-free imaging of vasculature and associated oxygen saturation makes PACT uniquely advantageous and enables multiple applications, ranging from clinical breast angiography [3,4] to pre-clinical whole-body imaging of small animals [9,10,11,12].

Currently, most PACT systems use Q-switched, Nd:YAG (neodymium-doped yttrium aluminum garnet)/OPO (optical parametric oscillator)-based, nanosecond pulsed tunable lasers and a multichannel ultrasound detector array with related data acquisition hardware [13,14,15,16,17]. These lasers typically deliver hundreds of mJ pulse energy to allow a sufficient photoacoustic signal-to-noise (SNR) ratio from targets situated several centimeters deep inside the tissue, such as deep vasculature of human breast. Because these lasers are expensive and bulky, their clinical use is limited. The high pulse energy lasers usually operate at low pulse repetition frequency (<20 Hz), and therefore can be traded with low pulse energy lasers that operate at high pulse repetition frequencies (>1 KHz) when the targeted application requires <2 cm imaging depth. Towards this goal, many researchers have employed both laser diodes [18,19,20,21,22,23,24] and light-emitting diodes (LED)s [24,25] for the development of low cost photoacoustic imaging systems. However, the emission from laser diodes still remains coherent and categorized as class-IV. Since the photoacoustic effect does not require light sources of high spatial coherence, LEDs have been explored as a safer and cost-effective alternative for tissue excitation, with additional advantages such as portability and ease of use. The state-of-the-art LED-based photoacoustic imaging setups typically attach two LED arrays on either side of a linear ultrasound array to enable real time B-mode photoacoustic (PA) combined with ultrasound (US) (LED–PAUS) imaging [26,27,28,29,30,31]. This includes employing dual-wavelength LED arrays, where individual LED elements of two different wavelengths are packed into a single array to map tissue oxygen saturation information. Each LED array emits approximately 200 µJ of pulse energy at 4 KHz pulse repetition frequency. This allowed generation of photoacoustic signals from mesoscopic depths inside the biological tissue. A sizable signal averaging is usually performed to improve the SNR, exploiting the high pulse repetition rates of LEDs, resulting in an effective frame rate of ~40 Hz. As such, these B-mode LED–PAUS systems lack high optical energy, multi-wavelength excitation, and volumetric imaging capabilities.

This work presents the first LED-based volumetric PACT system, referred to as the LED–PACT system from here onwards, capable of overcoming the limitations of B-mode LED–PAUS systems. The LED–PACT system employs multiple LED arrays and a linear ultrasound probe housed in a cylindrical structure that rotates 360 degrees to capture multiple projections through the object. While a linear scanning of the LED–PAUS imaging device across a phantom surface renders 3-D photoacoustic tomography (PAT) images by stitching of multiple 2-D PA frames, in our proposed LED–PACT, the 3-D image is obtained from rotational scanning around a cylindrical structure using time-reversal reconstruction. This configuration provides several advantages, such as higher and more uniform optical fluence distribution inside the tissue medium, overcoming the target shadowing problems, and multi-wavelength excitation to enable functional and molecular photoacoustic imaging capabilities similar to that of tunable lasers.

The rest of the paper is organized as follows. Section 2 describes the proposed LED–PACT system hardware and image reconstruction. The experimental design and the validation studies in different tissue-mimicking phantoms are presented in Section 3. The advantages and the limitations of the presented LED–PACT system and future directions that will help improve the speed and image quality are discussed in Section 4.

## 2. Materials and Methods

In this section, we first briefly describe the B-mode LED–PAUS system (AcousticX, Cyberdyne Inc., Ibaraki, Japan) that was adapted to develop the volumetric LED–PACT system. Next, the LED–PACT system hardware implementation and image reconstruction are described. 

### 2.1. B-Mode (LED–PAUS) System

**Figure 1:**
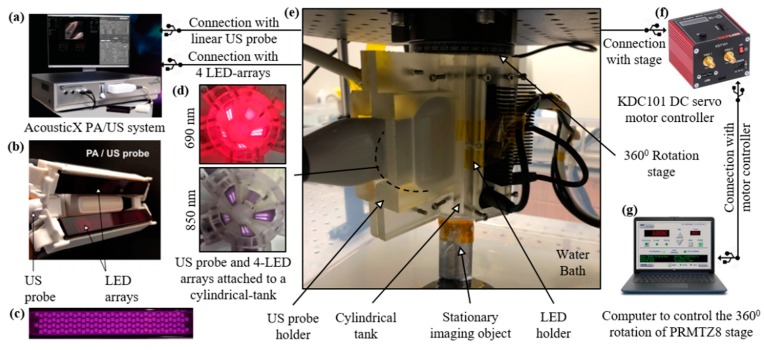
The experimental setup and schematic connections between different components of the light-emitting diode (LED)-based photoacoustic computed tomography (PACT) system. (**a**) The AcousticX system for combined photoacoustic/ultrasound (PA/US) data acquisition and for triggering four LED arrays. (**b**) Typical arrangement of the two LED arrays on either side of the US transducer device for B-mode PA/US imaging. (**c**) Picture of an LED array consisting of four rows of 36 LED elements with 1 mm × 1 mm dimensions. (**d**) Inside of a cylindrical tank showing the light emission from four dual-wavelength (690/850 nm) LED arrays and the emitting aperture of the US transducer array. (**e**) Photograph of our complete LED–PACT system housed in a cylindrical tank that is immersed in a water bath and attached to the rotation stage in the inverted configuration. (**f**) Picture of a motor controller for 360-degree rotation of the stage. (**g**) Computer giving control signals to the motor controller.

B-mode PA and US systems typically integrate optical fiber bundles, connected to a high-power and bulky tunable OPO laser, to a linear or curvilinear ultrasound transducer array [32]. In the AcousticX-based LED–PAUS systems (Figure 1a) two LED arrays are directly attached to a linear US transducer array (Figure 1b) for interleaved PA and US B-mode imaging. Each of these LED arrays consists of four rows of 36 1 mm × 1 mm LED elements (Figure 1c). For dual-wavelength LED arrays (e.g., 850 nm/690 nm), rows 1 and 3 are 850 nm, and rows 2 and 4 are 690 nm. These LED arrays are capable of delivering a maximum optical energy of 200 μJ per pulse and can be driven with a repetition rate of 1 KHz to 4 KHz and a pulse duration of 30 ns to 150 ns. The US transducer is a 128-element linear array having a pitch of 0.3 mm and total length of 38.4 mm. The central frequency of the transducer is 7 MHz and the measured −6 dB bandwidth is 75%. The ultrasound and photoacoustic modalities have sampling rates of 20 MHz and 40 MHz, respectively. The US transducer has an elevation focus of 15 mm, achieved with the help of an acoustic lens incorporated on the top of the transducer array.

### 2.2. LED-Based Photoacoustic Computed Tomography (LED–PACT) System

The experimental setup for the LED–PACT system is shown in Figure 1. A 3-D-printed cylindrical tank with an inner diameter of 38 mm, consisting of four slots for attaching four multi-wavelength LED arrays and one slot for a linear US transducer device, is used for assembling the components and subsequent imaging. Figure 1d shows the light emission inside of the tank from the four dual-wavelength 690/850 nm LED arrays. This imaging tank is attached to a rotational stage (PRMTZ8, ThorLabs Inc., Newton, NJ, USA) in the inverted configuration, as shown in Figure 1e. The rotational axis of the stage is aligned with the vertical axis of the cylindrical imaging tank. A tissue-mimicking phantom is kept stationary inside the imaging tank, such that it receives uniform light illumination during the rotational data acquisition. The imaging tank is immersed in a water bath to provide coupling between the ultrasound transducer and the imaging object or phantom. The DC servo motor controller (KDC101, ThorLabs Inc., Newton, NJ, USA), shown in Figure 1f, controls the rotational stage attached to the cylindrical tank with the help of a computer. 

The radio frequency (RF) scan mode of the AcousticX system is used to acquire the B-mode US and PA data at all rotational positions in synchronization, with the sequential triggering of multiple LED wavelengths. As described below, the acquired raw data is processed offline to reconstruct the volumetric photoacoustic images at each wavelength.

### 2.3. PACT Data Acquisition and Image Formation

The LED–PACT system acquires the raw PA data at a sampling rate of 40 MHz. One averaged PA frame is acquired using PA data from 2560 pulses at 4 KHz LED excitation rate. A total of 90 B-mode US and PA frames were acquired for each wavelength during the 360° rotation, with a 4-degree rotational step size and total scan time of <2 min. Each of the 128-transducer elements captures 1024 time samples. This results in a 90 × 1024 × 128 data matrix for 90 PA frames. A model-based time-reversal reconstruction algorithm [33] is applied, which numerically propagates the received PA pressure data back into the tissue medium from all the transducer elements. An Intel Xeon (2.1 GHz 32-core)-based computer with 128 GB RAM and Nvidia Titan Xp GPU was used for the reconstruction. Since the computation time of the model-based reconstruction methods increases exponentially with the size of the computational grid, two-dimensional computations can be orders of magnitude faster than three-dimensional computations. To be computationally efficient, we have applied the time-reversal algorithm in the 2-D plane formed by the rotation of a single transducer element. This is repeated for all 128-transducer elements individually, forming two-dimensional slices of the 3-D volume in 300 μm steps. The final 3-D image is formed by concatenating the 128 2-D slices into a single three-dimensional volume. With the above computational configuration, the reconstruction took five minutes for the entire 3-D reconstruction. 

## 3. Validation Experiments and Results

Here, we first present results from our simulation studies that compare the optical fluence distribution of the LED–PACT system with the standard B-mode LED–PAUS system. In the next sub-sections, we present experimental validation studies to test the structural, functional, and molecular imaging capabilities of our LED–PACT system. 

### 3.1. Optical Fluence Distribution of LED–PACT System

In order to measure the difference in optical fluence distribution between LED–PACT and LED–PAUS systems, we applied the finite difference method in MATLAB to solve the optical diffusion equation [34,35,36]:

∇·D(x) ∇Φ(x) + µ_a_(x) Φ(x) = 0, x ∈ X; Φ(y) = q(y), y ∈ ∂X (1)

In this equation, D = [3(μ_a_ + µ_s_’)]^−1^ is the diffusion coefficient, where μ_a_ = 0.1 cm^−1^ and μ_s_’ = 10.0 cm^−1^ are the absorption and reduced scattering coefficients of the simulated tissue medium [37]. Here, q(y) represents the optical source located at the boundary, either the LEDs or laser surrounding the region of interest; Φ(y) is the calculated fluence distribution due to the source q(y). As described in Section 2.1, the LED arrays used in this study consist of 1 mm x 1mm LED elements arranged in a 2-D matrix form (4 rows and 36 columns) with a 1 mm distance between the elements. The position of the LED elements of the array are defined as optical sources in a 3-D grid and the resulting optical fluence distribution inside a homogeneous tissue medium is calculated using the above diffusion equation. 

**Figure 2:**
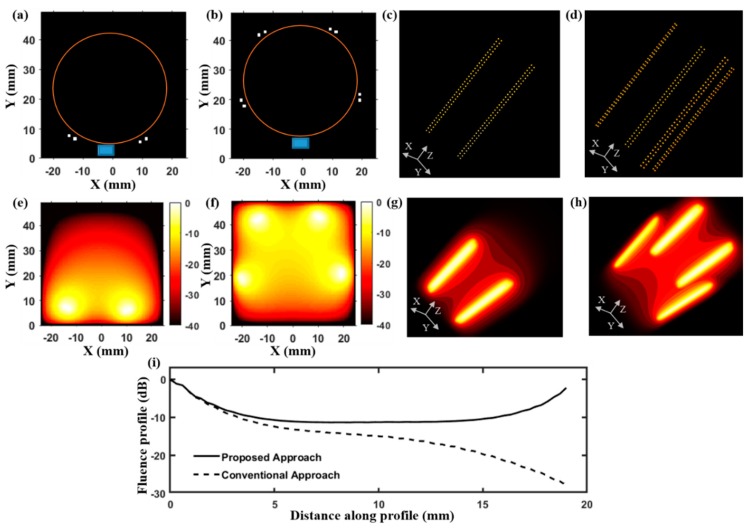
Comparison of optical fluence distribution inside the tissue medium of 5 cm diameter for the proposed LED-based PACT and the conventional B-mode PAUS systems. (**a**) Schematic of LED–PAUS geometry consisting of two 850/690 nm LED arrays (white dots) and a linear ultrasound transducer array (blue rectangle) placed on the boundary (orange circle) of the tissue medium. (**b**) A similar schematic for the PACT geometry shows the arrangement of four LED arrays with 72 degrees separation and the ultrasound transducer. (**c**,**d**) The corresponding 3-D schematic positions of the LED arrays in the PAUS and our PACT systems. Individual dots represent the positions of LED elements for a given wavelength in the dual-wavelength LED array. (**e**,**f**) A 2-D cross-section (at z = 20 mm) taken from the simulated 3-D fluence map inside the tissue medium for the two schematics shown in (**c**,**d**), respectively. (**g**,**h**) The 3-D fluence distribution for the two schematics shown in (**c**,**d**), respectively. (**i**) The fluence profile comparison at z = 20 mm along a diagonal in the imaging region of the PAUS geometry shown in (**c**) and the PACT illumination shown in (**d**).

Figure 2 presents a numerical simulation study that compares the optical fluence distribution inside a tissue medium resulting from the LED–PAUS illumination, which uses two LED arrays, and our LED–PACT illumination, which uses four LED arrays. Figure 2a,c shows 2-D and 3-D schematic arrangements of the LED–PAUS illumination consisting of two dual-wavelength 850/690 nm LED arrays placed on either side of the linear ultrasound transducer array, as in Figure 1b. Similarly, Figure 2b,d shows the 2-D and 3-D schematic arrangements of the PACT illumination using four LED arrays with 72 degree separation, and the linear ultrasound transducer array along the boundary of the cylindrical imaging tank (orange circle in Figure 2b). Figure 2e,f shows 2-D cross-sections (taken at z = 20 mm) of the 3-D simulated fluence inside a biological tissue medium for the PAUS and PACT illuminations, respectively. The corresponding 3-D optical fluence maps are shown in Figure 2g,h. To further study the difference in the optical fluence distribution between these two illumination geometries, in Figure 2i, we plot the magnitude (in dB) of the optical fluence in the X-Y plane at z = 20 mm along the diagonal of the imaging region containing the tissue medium.

### 3.2. Structural Imaging Studies

**Figure 3:**
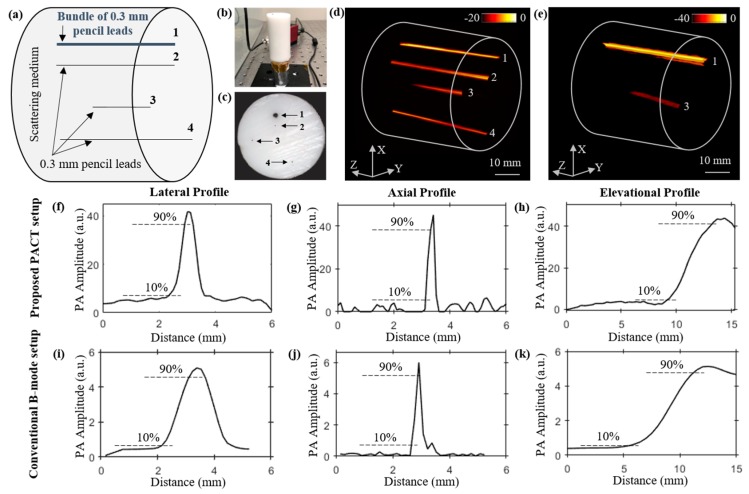
Comparing the structural imaging capabilities of the LED-based PACT and PAUS systems using a pencil lead phantom. (**a**) Schematic showing side view of a tissue-mimicking intralipid phantom with four targets embedded. The depth of targets from the top surface of the phantom are as follows: target-1 (bundle of five 0.3 mm pencil leads, at 10 mm), target-2 (0.3 mm pencil lead, at 14 mm), target-3 (0.3 mm pencil lead, at 23 mm), and target-4 (0.3 mm pencil lead, at 31 mm). (**b**,**c**) Photographs of the side and top views of the phantom. Reconstructed 3-D volume-rendered photoacoustic image (**d**) using PACT, and (**e**) linear scanning of the conventional PAUS systems. Photoacoustic amplitude plots of the pencil lead target-3, located at 23 mm depth inside the medium, along the lateral, axial, and elevational directions of the volume-rendered (**f**–**h**) PACT image shown in (**d**), and (**i**–**k**) the linear scan image shown in (**e**).

To study the structural imaging capabilities of our LED–PACT system, we imaged four pencil lead targets placed at different depths inside a tissue-mimicking phantom. The phantom is made of 1.5% agarose and 10% intralipid (INTRALIPID 20% IV Fat Emulsion, VWR international, Radnor, PA, USA) and has cylindrical dimensions of 35 mm diameter and 80 mm height. The optical absorption and reduced scattering coefficients of the phantom are expected to be 0.1 cm^−1^ and 10 cm^−1^, respectively [37]. Figure 3a–c shows the schematic view, a side view picture, and a top view picture of the phantom, respectively. The locations of the three pencil leads of 0.3 mm diameter each (marked as 2, 3 and 4 in Figure 3a), and a bundle of five pencil leads (marked as 1 in Figure 3a) inside the phantom can also be seen in these figures. The depth of these targets from the top surface of phantom are 10 mm, 14 mm, 23 mm, and 31 mm, respectively. The four targets are arranged such that target-2, with 0.3 mm diameter, is kept below (shadowed by) bundle target-1, with ~0.9 mm diameter. The PACT imaging of the phantom using the four LED arrays (850/690 nm pairs) and the 128-element ultrasound transducer array resulted in 90 B-mode PA frames during the 360 degree rotation of the phantom, with a 4 degree step size. In this lead phantom study, only the 850 nm wavelength of the LED arrays was switched on, yielding a total of 400 µJ pulse energy from the four LED arrays. Total time taken for the rotation (44.5 s for 89 rotations) and the data acquisition (57.6 s for 90 acquisition) was approximately 102.1 s. As shown in Figure 3d, the reconstructed 3-D volume-rendered PACT image obtained from the time-reversal algorithm applied on the 90 PA frames clearly displays the bundled lead target-1 and three other pencil leads targets.

We further compare these volumetric PACT images with the 3-D PAT image (Figure 3e) obtained from the linear scanning of the phantom using the LED–PAUS system, which uses the 850 nm wavelength of the two LED arrays and the same ultrasound device, as shown in Figure 1b. Only bundled pencil lead target-1 and target-3 are seen in the reconstructed image in Figure 3e. This is because the smaller photoacoustic target-2, located behind a thicker photoacoustic target-1, is likely shadowed during the linear scanning of the PAUS system. The same target-2 is clearly imaged by the PACT as it employs the rotational scan for volumetric imaging. Target-4, located 31 mm below the phantom surface, is not visible in the linear scan of the conventional LED–PAUS imaging because of the following reason. To achieve uniform illumination on the tissue surface, the imaging head (two LED arrays attached to the ultrasound device) is placed ~10 mm from the tissue surface. This 10 mm standoff region is filled with the ultrasound coupling medium (water in our case). This reduces the effective imaging depth to only 30 mm inside the tissue medium. Since the AcousticX-based data acquisition is only limited to 40 mm depth of imaging, target-4 is not visible. The PACT system overcomes this limitation because (1) uniform light illumination on the surface is achieved with no 10-mm standoff region, and (2) all four targets appear within the 40 mm depth of view during the rotation scan of the system around the phantom. 

We further compared the spatial resolutions of the PACT and PAUS systems using the respective volume-rendered images shown in Figure 3d,e. Figure 3f–h and Figure 3i–k, respectively, show the photoacoustic intensity plots of the pencil lead target-3 along the lateral, axial, and elevational distances of the two images shown in Figure 3d,e. In these plots, half of the distance between 10% and 90% of the peak photoacoustic amplitude is estimated as the spatial resolution. These calculations show that the lateral, axial, and elevational resolutions of the PACT imager are ~300 μm, 120 μm, and 2.1 mm, respectively; and for the PAUS system, the resolutions are 600 μm, 130 μm, and 3 mm, respectively.

### 3.3. Multispectral Molecular LED–PACT

In this section, we validate the multispectral photoacoustic imaging capabilities of the LED–PACT system by employing two dual-wavelength 850/690 nm LED arrays and two 470 nm LED arrays. Each of these four LED arrays placed around the cylindrical tank can be selectively switched “ON” or “OFF” to sequentially excite the tissue with 850 nm, 690 nm, or 470 nm wavelengths at each rotational angle, with a 4 degree step size, during the 360 degree rotation around the phantom. This resulted in acquisition of 90 PA frames for each of these wavelengths. 

**Figure 4:**
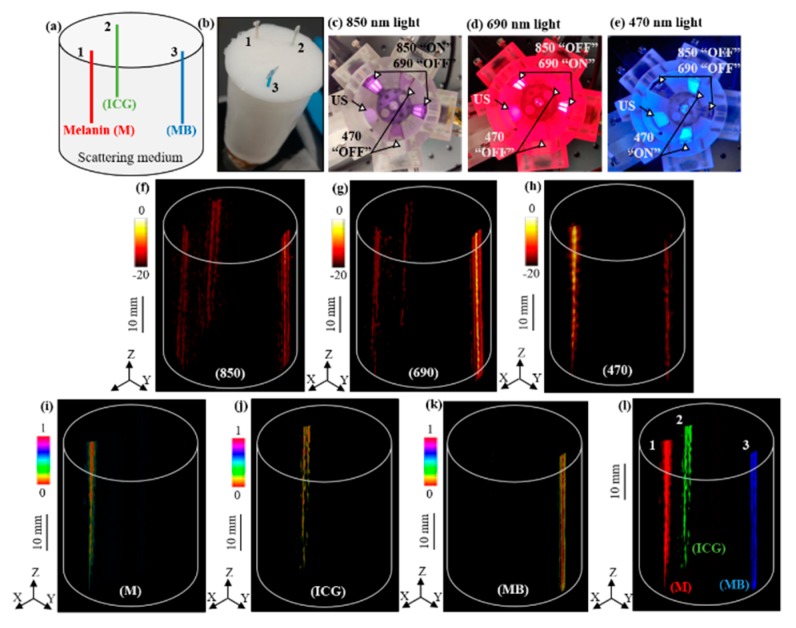
LED-based multispectral photoacoustic computed tomography. (**a**) Schematic view and (**b**) a photograph of the tissue-mimicking cylindrical phantom with 35 mm diameter and 80 mm height. The phantom is embedded with 0.5 mm polyethylene tubes filled with 1 mM concertation solutions of melanin (M), indocyanine green (ICG), and methylene blue (MB). (**c**–**e**) The arrangement of THE ultrasound (US) transducer array, two 850/690 nm LED arrays, and two 470 nm LED arrays with sequentially switched 850 nm, 690 nm, and 470 nm wavelength emissions from the arrays. (**f**–**h**) The 3-D PACT images of the phantom acquired with 850 nm, 690 nm, and 470 nm illumination, respectively. (**i**–**k**) Spectrally unmixed volumetric images of the phantom, obtained from the multispectral PA images in (**f**–**h**), showing the spatial distribution of M, ICG, and MB tubes, respectively. (**l**) Superimposed 3-D spectrally unmixed image of the three chromophores. Scale bar is 10 mm in images (**f**–**l**).

We validated our approach using a tissue-mimicking intralipid phantom consisting of 0.5 mm diameter polyethylene tubes filled with three different light absorbing molecular solutions of 1 mM concentration, namely indocyanine green (ICG), methylene blue (MB), and melanin (M), having higher optical absorptions around 790 nm, 680 nm, and 500 nm wavelengths, respectively. Figure 4a,b shows the schematic positions of the three tubes inside the phantom and a photograph of the phantom. Figure 4c–e shows the arrangement of the four LED arrays with respect to the US transducer device inside the cylindrical imaging tank, which is sequentially triggered to provide 200 μJ of pulse energy for each of the 850 nm, 690 nm, and 470 nm wavelengths, respectively.

Figure 4f–h shows the reconstructed volumetric PACT images of the above phantom using the 90 PA frames acquired at each of the 850 nm, 690 nm, and 470 nm wavelengths, respectively. As shown in Figure 4i–k, linear spectral unmixing of the multispectral PA images (Figure 4f–h) clearly reveals the melanin, ICG, and MB tubes. We further superimposed the three spectrally unmixed images in Figure 4l to confirm the respective volumetric distribution of the three chromophores.

### 3.4. Functional Oxygen Saturation Imaging Using a Human-Finger-Mimicking Phantom

**Figure 5:**
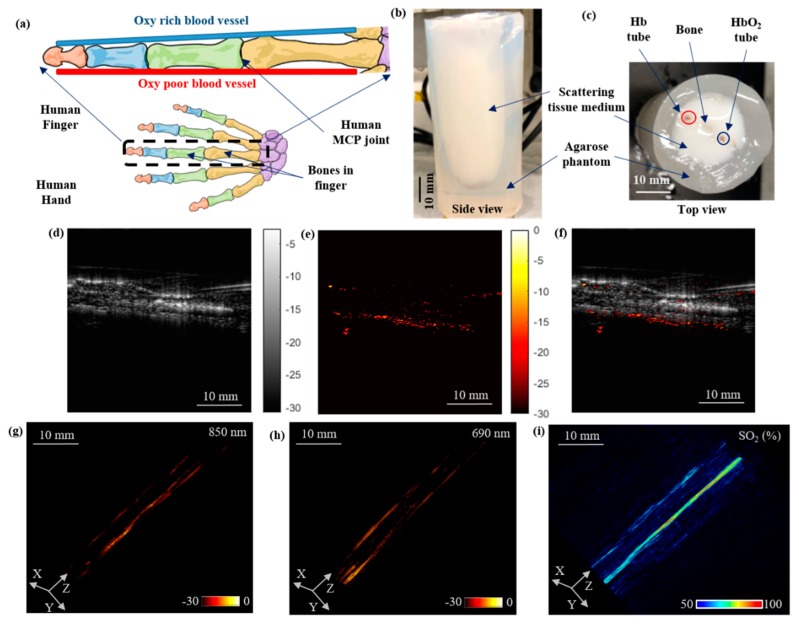
LED–PACT-imaging of vascular oxygen saturation using a human-finger-mimicking phantom. (**a**) Schematic sketch of a typical human finger with the location of bones and the blood vessels (oxy rich: HbO_2_; oxy poor: Hb). (**b**,**c**) The side and the top view photographs of the finger phantom consisting of bone and blood vessels. (**d**) The ultrasound (US), (**e**) photoacoustic (PA), and (**f**) co-registered PA–US frames acquired at one of the rotational steps during the 360 degree rotation around the phantom. (**g**,**h**) The reconstructed 3-D volume-rendered PACT images of the finger phantom using 850 nm and 690 nm LED illuminations, respectively. (**i**) The spectrally unmixed volumetric oxygen saturation map for the finger phantom.

In our final experimental validation, we demonstrated the vascular oxygen saturation imaging capabilities of the PACT system using a human-finger-mimicking, highly scattering (μ_s_’ = 15 cm^−1^), intralipid phantom consisting of 0.5 mm polyethylene tubes filled with oxygenated-(saturated with oxygen) and deoxygenated-(saturated with carbon dioxide) bovine blood (Bovine Blood CITR, Carolina Biological Supply, Charlotte, NC, USA) placed on the either side of an animal bone (Figure 5a). The side and top view photographs of the phantom are shown in Figure 5b,c, respectively.

The PACT experimental setup used to image this phantom consisted of four 850/690 nm LED array pairs attached to the cylinder tank, as shown in Figure 1d. At each rotation step of 4 degrees, two different PA frames were acquired in synchronization with the sequentially switched 850 nm and 690 nm wavelengths. Figure 5d–f represents the US, PA (at 850 nm), and co-registered (PA–US) frames at a single rotation step, captured during the full tomography acquisition. The structure of the finger is visible in the US image and the optical absorption contrast of the blood tubes is mapped in the PA frame. The volume rendered PACT images at 850 nm and 690 nm, obtained from the time-reversal reconstruction of 90 PA frames acquired at each of these wavelengths, are shown in Figure 5g,h, respectively. Figure 5i shows the volumetric oxygen saturation map of the finger phantom obtained using spectral unmixing of the PACT images at 850 nm and 690 nm wavelengths.

## 4. Discussion

A low-cost and portable PACT system was developed using multi-wavelength LED arrays as optical sources and a linear ultrasound transducer array for the photoacoustic detection. The PACT system is capable of rotating 360 degrees around an imaging object in a cylindrical shape and acquires 2-D US as well as PA data of multiple wavelengths at each rotational angle. Because LEDs usually output low pulse energies, a sizable PA frame averaging is performed to achieve a decent signal-to-noise ratio, exploiting the high pulse repetition rates of LEDs. The 3-D PACT images are formed using time-reversal reconstruction of the acquired PA data. The total scan time for full tomography took around 102 s for each wavelength and about five minutes for the image reconstruction using the time-reversal algorithm. 

While B-mode LED–PAUS systems can only use two LED arrays, the proposed LED–PACT system geometry is capable of employing more than the four LED arrays used in this study. This not only increases the optical energy density inside the tissue medium, but also allows custom integration of multi-wavelength LEDs suitable for spectroscopic photoacoustic imaging. The volumetric structural, functional, and molecular imaging capabilities of the LED–PACT system is validated using different types of tissue-mimicking phantoms.

First, the pencil-lead phantom imaging experiments demonstrated that the PACT system can see through the shadow (blind spot) imaging regions of the conventional PAUS systems and visualize smaller targets hiding behind larger targets. These experiments also demonstrated that the spatial resolutions of the PACT system are better than the PAUS. These advantages can be attributed to the fact that the PACT system enables more uniform illumination of the imaging region during the 360 degree rotation. The conventional LED–PAUS systems require the imaging head to be 10 mm above the tissue surface to achieve uniform illumination of the phantom. This 10 mm standoff is usually filled with ultrasound coupling medium and leads to several complications, such as (1) creation of bubbles and associated artifacts during the linear scan, (2) ultrasound and optical attenuation inside the thick coupling medium, (3) uncomfortable imaging of living subjects, and (4) extended imaging depth and computer memory, which reduces imaging speed. The LED–PACT system demonstrated here required no such stand-off, and therefore could image all 4 pencil lead targets inside the phantom, whereas the LED–PAUS system missed the target-4 at 31 mm depth.

One of the main advantages of our LED–PACT system compared to the existing LED-based PA system is that it can be easily adapted to allow custom designed multi-wavelength excitation using multiple LED arrays to enable similar functional and molecular imaging capabilities as tunable lasers. This is demonstrated by imaging a tissue-mimicking phantom embedded with tubes of melanin, ICG, and MB solutions using two dual-wavelength 850/690 nm LED arrays and two 470 nm LED arrays. Since the optical absorption of melanin decreases with increase in the optical wavelength, the PA contrast of the melanin is higher at 470 nm and lower in the 870 nm PACT images. Similarly, the peak absorption of MB at ~680 nm correlates well with the highest PA intensity of the MB tube at the 690 nm wavelength. Based on these spectral trends, the linear spectral unmixing technique could easily separate the three chromophores with the PACT data acquired at 470 nm, 690 nm, and 850 nm optical wavelengths. 

We further validated the vascular oxygen saturation imaging capabilities of the LED–PACT system by imaging a human-finger-mimicking phantom using four dual-wavelength 850/690 nm LED arrays. This configuration delivered higher (400 µJ) pulse energy for 850 nm as well as 690 nm wavelengths than possible with the conventional LED–PAUS systems, which can accommodate only two such LED arrays. This enabled mapping of vascular oxygen saturation from deeper regions.

This current study was designed to demonstrate the abovementioned various advantages of our novel LED–PACT system using proof-of-concept experiments on tissue-mimicking phantoms. We plan to further improve the imaging performance of the LED–PACT system from multiple directions, including a better imaging shape that employs more than 6 LED arrays, faster data acquisition, model-based image reconstruction algorithms [38,39,40], and deep learning approaches [41,42]. A fully developed LED–PACT will then be validated on living subjects, for example imaging small animals and human body parts, such as fingers, wrists, and breasts. 

Despite these limitations, we believe these experimental studies demonstrate that our LED-based PACT system has strong potential to become a powerful biomedical imaging tool for resource-limited pre-clinical and clinical settings.

## 5. Conclusions

We have developed a LED-based PACT system for biomedical applications. The system integrates four LED arrays and a linear ultrasound transducer array in a cylindrical housing. A 360-degree rotation of the system around the stationary imaging object provides volumetric photoacoustic images using the time-reversal reconstruction algorithm. The LED–PACT system has several benefits compared to the existing LED-based PAUS systems, such as multispectral photoacoustic imaging, better spatial resolution, uniform illumination, and improved imaging depth. The axial, lateral, and elevational resolutions of the PACT system were estimated as 120 µm, 300 µm, and 2.1 mm, compared to 130 μm, 600 μm, and 3 mm for the B-mode. Validation experiments on different tissue-mimicking phantoms demonstrated the structural, functional, and molecular photoacoustic imaging capabilities of the system. This included imaging of light absorbing chromophores (melanin, ICG, methylene blue, oxygenated hemoglobin, and deoxygenated hemoglobin) inside a tissue-mimicking phantom using multi-wavelength LED arrays. With further optimization, such as increase in the number of LED arrays and model-based image reconstruction, LED-based PACT imaging systems could be promising biomedical imaging tools for use on living subjects.

## Abbreviations

The following abbreviations are used in the manuscript:

PA	Photoacoustic
US	Ultrasound
PAI	Photoacoustic Imaging
PACT	Photoacoustic Computed Tomography
LED	Light Emitting Diode
